# Intelligent ground vibration prediction in surface mines using an efficient soft computing method based on field data

**DOI:** 10.3389/fpubh.2022.1094771

**Published:** 2023-02-01

**Authors:** Behrooz Keshtegar, Jamshid Piri, Rini Asnida Abdullah, Mahdi Hasanipanah, Mohanad Muayad Sabri Sabri, Binh Nguyen Le

**Affiliations:** ^1^Department of Civil Engineering, Faculty of Engineering, University of Zabol, Zabol, Iran; ^2^Department of Water Engineering, Faculty of Water and Soil, University of Zabol, Zabol, Iran; ^3^Department of Geotechnics and Transportation, Faculty of Civil Engineering, Universiti Teknologi Malaysia, Johor Bahru, Malaysia; ^4^Institute of Research and Development, Duy Tan University, Da Nang, Vietnam; ^5^Peter the Great St. Petersburg Polytechnic University, St. Petersburg, Russia; ^6^School of Engineering and Technology, Duy Tan University, Da Nang, Vietnam

**Keywords:** blasting, ground vibration, hybrid soft computing method, RSM, SVR

## Abstract

Ground vibration induced by blasting operations is considered one of the most common environmental effects of mining projects. A strong ground vibration can destroy buildings and structures, hence its prediction and minimization are of high importance. The aim of this study is to estimate the ground vibration through a hybrid soft computing (SC) method, called RSM-SVR, which comprises two main regression techniques: the response surface model (RSM) and support vector regression (SVR). The RSM-SVR model applies an RSM in the first calibrating process and an SVR in the second calibrating process to improve the accuracy of the ground vibration predictions. The predicted results of an RSM, which are obtained using the input data of problems, are used as the input dataset for the regression process of an SVR. The effectiveness and agreement of the RSM-SVR model were compared to those of an SVR optimized with the particle swarm optimization (PSO) and genetic algorithm (GA), RSM, and multivariate linear regression (MLR) based on several statistical factors. The findings confirmed that the RSM-SVR model was considerably superior to other models in terms of accuracy. The amounts of coefficient of determination (*R*^2^) were 0.896, 0.807, 0.782, 0.752, 0.711, and 0.664 obtained from the RSM-SVR, PSO-SVR, GA-SVR, MLR, SVR, and RSM models, respectively.

## 1. Introduction

**Figure 1:**
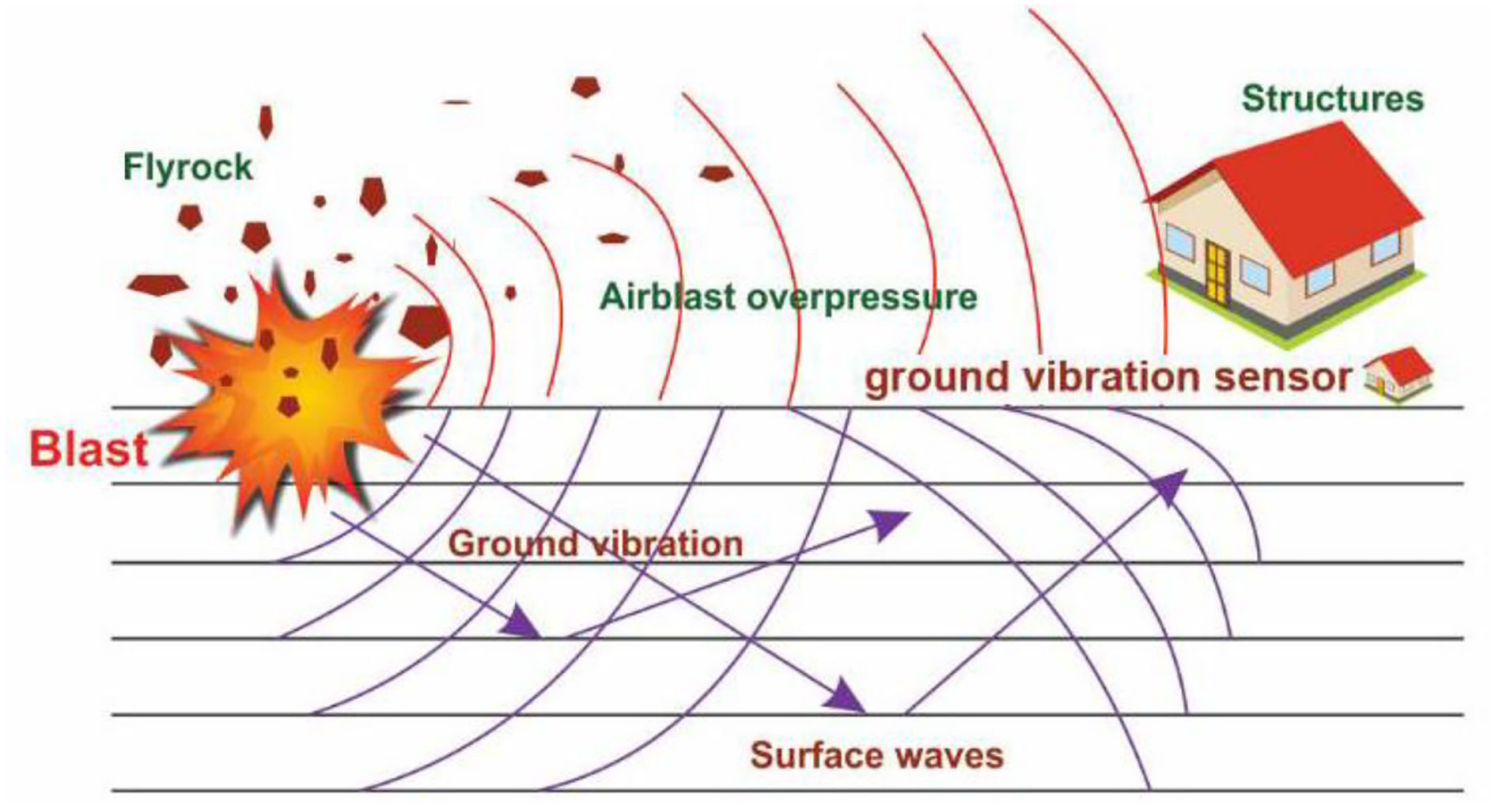
Phenomena induced by blasting (33).

When excavating hard rock (which is required specifically in mining and quarrying operations, and generally when constructing highways, subways, tunnels, and dams), a common activity is drilling and blasting operations. As confirmed in the relevant literature, blasting unavoidably leads to some adverse impacts such as air blasts, ground vibrations, back breaks, flyrocks, and noise (Figure 1) (1–11). Although it is quite impossible to entirely eliminate all these impacts, they can be minimized. Ground vibration, among all, is one of the most important concerns in this sense (12–14). The extent of vibration occurred to a given structure is dependent upon different parameters such as the method of construction, distance from the source, soil/rock medium, heterogeneity of the soil and rock deposit, features of the waves propagated at the given site, the structure's susceptibility rating, the soil/rocks dynamic features, and the fracture's response characteristics (15, 16). Most of these parameters (particularly those related to geotechnical and geological conditions) are not controllable; however, the amount of explosive material and other blast design parameters such as burden (B), spacing (S), and powder factor (PF) can be controlled (17). The literature comprises different studies focusing on how to decrease the environmental impacts induced by blasting operations; though, due to the complexity of the problem, it lacks a general consistent approach or a certain formula in this regard. Not only the wave and ground motion features but also the complexity of blasting parameters and site factors has limited the scholars working in this field. This hinders the effective development of a widely-accepted criterion for measuring the geological parameters and blasting data and also ensuring the serviceability of susceptible constructions (18). It is possible to control the extent of ground vibration by choosing appropriate blasting methods and the best drilling/firing pattern. Generally, the vibration source produces body and surface waves in the rock/soil medium (16). Body waves are propagated through the rock and soil deposits. The most important types of body waves are compression and shear waves which need to be well considered at a comparatively small distance from the construction sources. On the other hand, the surface waves (whose main type is Rayleigh waves) are normally propagated along the upper surface of the ground. As building foundations are typically positioned near the ground surface, the Rayleigh waves usually attract great attention from structural engineers.

Peak particle velocity (PPV) and frequency are the most important descriptors in measuring blast-induced ground vibration. Among them, the former has been more widely used in previous studies (9–11). Accordingly, PPV is used in this study to measure ground vibration. The maximum charge used per delay (Mc) and distance from the blasting point (Di) (which have been used in various empirical approaches) are the two main parameters to calculate the PPV value. For example, Ghosh and Daemen (19), Gupta et al. (20, 21), and Roy (22) have presented several empirical approaches in this regard. In general, different factors are considered in one excavation site with the aim of testing the velocity equations based on actual field measurements, which finally results in different PPV values against the safe *MC* (23). To apply the controlled blasting techniques and to specify the relevant site-specific constants, many blasting data should be collected from the adjacent region (24, 25). In real conditions, the collected data are sometimes widely distributed and have a low correlation coefficient. In such cases, the equations obtained based on the assessment of these data typically demonstrate a low reliability level. Such unreliability is because of the variation in site-specific constants with direction (26, 27).

Recently, numerous soft computing (SC) methods have been introduced across the world. These tools help to find more accurate and authenticated solutions to complex problems that appear in engineering contexts. Such tools (28–34) have been developed and implemented by various scholars and practitioners working in different fields such as mining, civil engineering, geoengineering, and mechanics.

The prediction of blast-induced PPV has been done using several methods such as SC-based models. Ghasemi et al. (5) attempted to develop empirical models and a fuzzy model (FM) in order to predict the PPV value. To evaluate the model with the optimum performance, a number of performance indices, e.g., coefficient of determination (*R*^2^), were proposed. According to their findings, an FM was capable of predicting PPV with a higher accuracy level in comparison with empirical equations. The FM recorded the *R*^2^ value of 0.94, while for the empirical equations, this value was recorded as only 0.65. Furthermore, Radojica et al. (35) utilized empirical equations and an artificial neural network (ANN) to predict the PPV value. The final results showed a higher accuracy of the ANN, with *R*^2^ = 0.91, compared to the empirical methods. To evaluate PPV, Hajihassani et al. (6) proposed a hybridization of an ANN with the imperialist competitive algorithm (ICA). Their results showed the *R*^2^ values of 0.97, 0.91, and 0.87, obtained from the ICA-ANN, ANN, and MR models, respectively, which obviously indicates the higher effectiveness of the ICA-ANN compared to the other models. PPV in the study of Hasanipanah et al. (36) was estimated with the use of a genetic algorithm (GA). In separate studies, Hasanipanah et al. (37) and Jahed Armaghani et al. (7) made use of particle swarm optimization (PSO) and ICA, respectively, for the same objective, i.e., estimating the PPV value. Their findings showed the effectiveness of the GA, ICA, and PSO models in terms of developing non-linear equations applicable to the PPV prediction. The ANN performance was improved by Jahed Armaghani et al. (38, 39) and Hasanipanah et al. (40) by integrating it with optimization tools such as PSO. Taheri et al. (41) integrated an ANN with the artificial bee colony (ABC) algorithm as a way to estimate PPV. The comparative results showed the high capacity of an ABC in improving the performance quality of an ANN. Shahnazar et al. (42) predicted PPV by integrating PSO with a neuro-fuzzy system. Their findings revealed the superiority of the hybrid model over the neuro-fuzzy system regarding PPV prediction. In Hasanipanah et al.'s (43) study, an ICA was combined with the fuzzy system (FS) and it was found successful in estimating the PPV value. In another research, Nguyen et al. (44) tested the random forest (RF) and extreme gradient boosting (XGBoost) models in terms of their effectiveness in estimating PPV. Findings confirmed that the XGBoost model was more successful than the rival regarding the defined task. In recent years, the Gaussian process regression (GPR) was investigated by Arthur et al. (8) regarding its accuracy in predicting ground vibration. According to their results, GPR had a higher capacity compared to empirical models in predicting PPV.

Recently, a novel SC approach was proposed by Zhang et al. (45) with the same objective. They utilized PSO in order to optimize the XGBoost. According to the results, if the PSO is effectively integrated with the XGBoost, the model's performance can be meaningfully improved. The boosted generalized additive models (BGAMs) were introduced by Nguyen et al. (10) with the aim of predicting PVV. They compared the results of their models with those of an ANN and a support vector machine (SVM). BGAMs were found practical and effective with results better than those of the ANN, SVM, and empirical models. Fang et al. (11), on the other hand, made use of an ICA for the purpose of optimizing the M5Rules model in estimating the PPV value. The findings confirmed that the ICA-M5Rules model was more successful than the conventional M5Rules, SVM, and RF models with regard to predicting PPV. Chandrahas et al. (46) used the K-Nearest Neighbor, XGBoost, and Random Forest models to predict PPV, and showed the effectiveness of XGBoost in this field compared to two other models. The gray wolf optimizer (GWO), as an optimization algorithm, was combined with an extreme learning machine (ELM) by Yan et al. (32) to predict PPV. They concluded the hybrid method was more effective and robust than the ELM and empirical models. The mentioned optimization algorithm was also combined with the relevance vector regression (RVR) with the same aim by Fattahi and Hasanipanah (33). For comparison purposes, a bat-inspired algorithm-RVR was used. The results confirmed that GWO-RVR performed better than the bat-inspired algorithm-RVR, which proved the effectiveness of GWO to improve the RVR model. In another hybrid model, a combination of an ANN and a Hunger Games Search (HGS) algorithm was tested by Nguyen and Bui (34) in terms of PPV prediction. In their study, three other optimization algorithms were also employed, and according to their results, the ANN-HGS model achieved more satisfactory predictive performance than the other models.

Zhang et al. (47) predicted PPV using chi-squared automatic interaction detection (CHAID), an RF, an ANN, an SVM, and classification and regression trees (CART). According to their results, the SVM yielded better performance for the prediction of PPV compared to others. Jahed Armaghani et al. (48) predicted PPV using a least square–SVM (LS–SVM), a GPR, a minimax probability machine regression (MPMR), and a PSO-extreme learning machine (PSO-ELM). The results indicated that the PSO-ELM was more computationally efficient with better predictive ability. Table 1 lists some studies in the field of PPV prediction.

**Table 1:** Some studies in the field of PPV prediction.

**References**	**SC technique**	**Input parameters**
Khandelwal et al. (49)	SVM	Mc, Di
Dindarloo (50)	GEP	B, S, St, Mc, Di, radial distance, number, depth and diameter of holes
Saadat et al. (51)	ANN	Mc, Di, S, bench height
Taheri et al. (41)	ABC-ANN	Mc, Di
Mokfi et al. (52)	GMDH	B/S, hole depth, St, PF, Mc, Di
Azimi et al. (53)	GA-ANN	Mc, Di, radial distance, modified radial distance
Bui et al. (54)	PSO-KNN	Mc, Di
Chen et al. (55)	MFA-SVR	Mc, Di, B/S, St, E, Vp
Hasanipanah et al. (43)	FS-ICA	Mc, Di
Xue (56)	FCM-ANFIS	Mc, Di, scaled distance
Fang et al. (11)	ICA-M5Rules	Mc, Di, B, S
Hajihassani et al. (6)	ICA-ANN	B/S, St, Mc, Di, E
Amiri et al. (57)	ANN-KNN	Mc, Di
Sheykhi et al. (58)	FCM-SVR	B, S, St, number of holes per delay, Mc, Di
Yu et al. (59)	RVM	Mc, Di, B, vertical distance, Protodyakonovs impact strength coefficient, total explosive charged, delay time of detonator
Nguyen et al. (60)	GA-SVR-RBF	Mc, Di, B, S
Ding et al. (61)	ICA-XGBoost	Mc, Di, bench height, S, St, powder factor, B
Nguyen et al. (9)	HKM-CA	Mc, Di, bench height, powder factor, St, B, S
Zhou et al. (62)	FS-RF	S, B, Mc, Di, hole depth

B/S, burden to spacing ratio; St, Stemming; Vp, p-wave velocity; E, Young modulus; GEP, genetic expression programming; GMDH, group method of data handling; ANFIS, adaptive neuro-fuzzy inference system; KNN, k nearest neighbors; SVR, support vector regression; MFA, modified firefly algorithm; FCM, fuzzy Cmeans clustering; RBF, radial basis function; RVM, relevance vector machine; HKM-CA, hierarchical K-means clustering-cubist algorithm.

In this study, the blast-induced PPV is predicted using a hybrid SC approach, called RSM-SVR, comprising two main regression techniques: the response surface model (RSM) and support vector regression (SVR). The proposed RSM-SVR model is structured using the RSM in the first calibrating process and SVR in the second calibrating process to improve the accuracy of the blast-induced PPV predictions. Then, the accuracy of the RSM-SVR model is compared with that of SVR, the RSM, and multivariate linear regression (MLR) based on several statistical factors. In addition, the PSO and GA, as two optimization algorithms, were employed and compared with the other models. The main contribution of this study to the body of knowledge is to propose a novel and efficient hybrid SC model, namely RSM-SVR, applicable in predicting blast-induced PPV.

The rest of this article includes the following. More details about the source of the datasets and also the hybrid SC model are explained in the second section. The results and discussions are provided in the third and fourth sections; then, the last section presents the conclusions of the study.

## 2. Materials and methods

### 2.1. Materials

The data used in this study were collected from the Harapan Ramai granite quarry located in the northern part of Johor, Malaysia. This quarry produces ~35,000–40,000 tons of granite aggregates per month. During each month, 8–10 blasting operations (depending on the weather condition) are performed. The main initiation and explosive materials are dynamite and ANFO, respectively. Fine gravel is used for the purpose of stemming the blast holes required.

For the purpose of this study, the parameters of *MC, B, S*, and *St* were measured before each blasting operation. Moreover, the block samples corresponding to each blast were transferred to the laboratory in order to measure the *Vp* and the unconfined compressive strength (*UCS*), according to ISRM (63). Based on the dataset collected, the burden-to-spacing ratio (*B/S*), *St, MC, E, Vp*, and *Di* were set as input variables.

In each blasting, PPV was recorded using the VibraZEB seismograph which possesses certain transducers for measuring PPV. In total, 90 blasts were monitored and the PPV in each event was calculated. The statistical characteristics of training and testing data sets for input variables are listed in Table 2. As indicated by the skewness results, the skewness does not follow the zero values for all data and almost all the variables tended to non-normal distributions. This means that the relations between PPV and almost all the input data can be described based on the non-linear form. As can be seen in Table 2, the skewness, STD, and mean of the database in the testing and training phases are assigned with different values for each variable of the input data set and PPV. The robustness and accuracy of predictions are more vital subjects for the validation of a model, which are learned using the data set in the training phase. The ability of a model to predict the non-linear relation is important in the current complex engineering problem. Thus, four non-linear models, i.e., MLR, RSM, SVR, and the proposed RSM-SVR are compared in terms of estimating PPV based on six input variables.

**Table 2:** Statistical characteristics of data sets.

**Variables**	**Train phase**						**Test phase**					
	**X**_min_	**X**_max_	**Mean**	**STD**	**COV**	**Skewness**	**X**_min_	**X**_max_	**Mean**	**STD**	**COV**	**Skewness**
Mc (kg)	133.59	534.37	345.970	131.387	0.380	−0.204	534.37	634.04	584.5004	28.776	0.049	−0.240
B/S	0.79	0.89	0.828	0.025	0.030	0.485	0.79	0.87	0.833	0.031	0.038	−0.026
St (m)	1.5	7	4.392	1.667	0.379	−0.244	7	8	7.32	0.379	0.052	0.688
E (GPa)	8.41	29.886	24.887	4.669	0.188	−1.877	19.606	29.748	24.965	3.220	0.129	−0.014
Vp (m/s)	2,805	4,506	3,769.446	525.157	0.139	−0.271	2,809	4,344	3,560.960	483.006	0.136	0.145
Di (m)	90	440	281.169	104.861	0.373	−0.560	195	430	334.040	62.618	0.187	−0.455
PPV(mm/s)	2.834	33.08	14.193	7.811	0.550	0.829	5.74	19.23	13.550	4.890	0.361	−0.400

### 2.2. Methods

**Figure 2:**
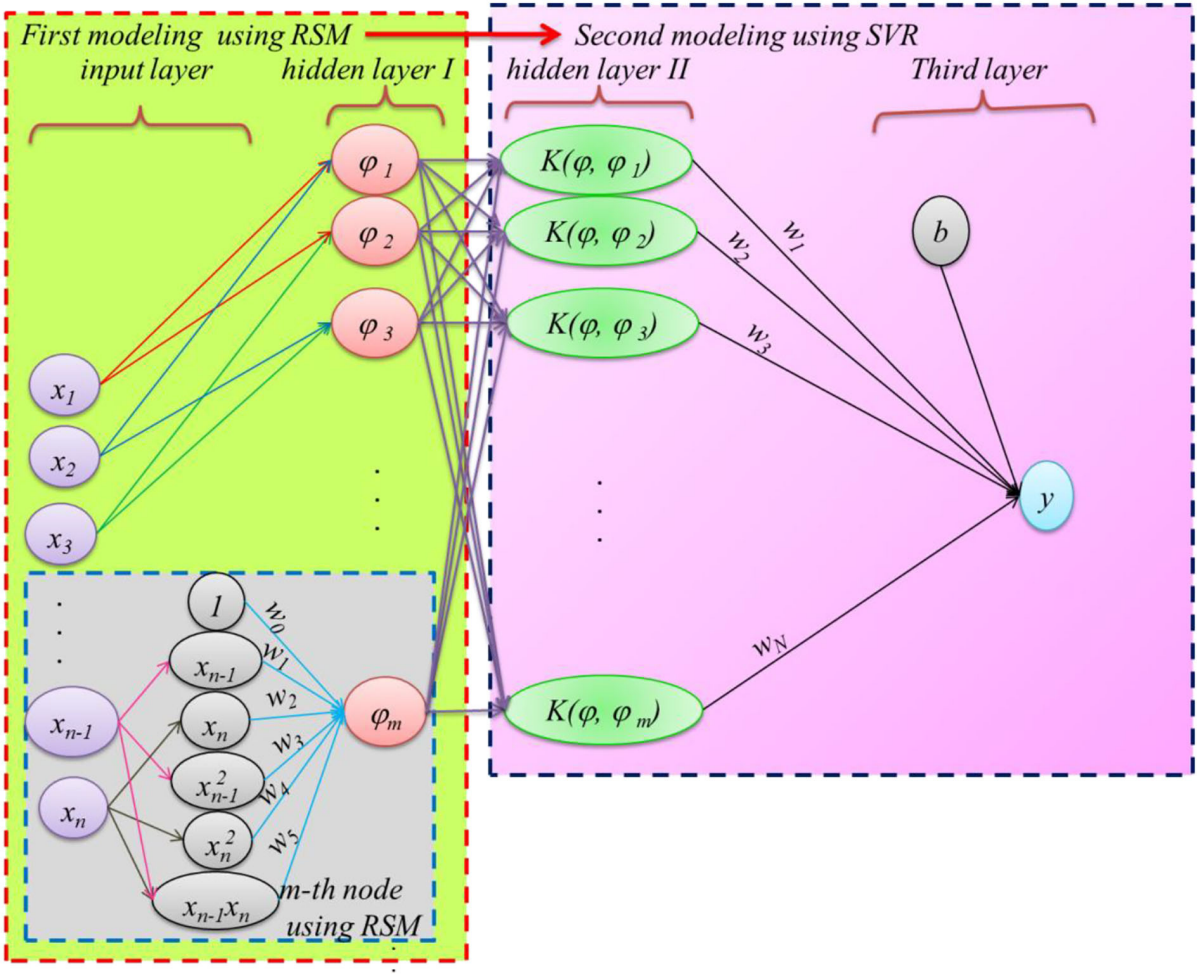
A view of RSM-SVR.

To achieve an accurate prediction of PPV, a novel SC method is herein proposed by integrating RSM with SVR models, i.e., RSM-SVR. The non-linear relation may be provided based on two regression processes of RSM and SVR between PPV and input factors. In this regard, RSM is combined with SVR based on two calibrating processes through which the input data of the proposed ML model is provided based on the RSM predictions. The structure of the hybrid RSM as input and SVR as an ML scheme (RSM-SVR) is plotted in Figure 2. As can be seen in this figure, the proposed RSM-SVR has three basic layers: (i) the input layer, (ii) the hidden layer [which is separated into two main layers, i.e., (1) predicted data set using RSM, and (2) the input data set using predicted data by RSM to provide the feature data in SVR], and (iii) the predicted layer using SVR. It is propsed that the input data set for SVR is provided based on the non-linear RSM relations that are predicted based on two individual input variables.

The input data sets, which are used to calibrate the response surface polynomial functions, should be selected with different input variables in the first calibrating process. Therefore, *m*–input data were prepared to calibrate the SVR provided based on the RSM by using *n*–input data. Thus, for the SVR model, *n*-dimension variables are transferred into *m*-dimension as follows:


(1) $$\begin{array}{l} m=\frac{n!}{2!\times \left(n-2\right)!} \end{array}$$

where ! is the factorial operator. This model involves two main regression procedures: RSM and SVR. The former is used in the first stage, which can be extracted from the input data as presented in Figure 2. The *m*–input data provided in the hidden layer is computed using an RSM, which is a second-order polynomial function with cross-terms and two input variables (64, 65). Therefore, a simple non-linear relation is applied to estimate the *m*–hidden node using the RSM. The SVR prediction in the proposed method is dependent on the *m*–input data set. The input database-based estimated RSM is represented by a non-linear map ϕ_*n*_ with weights *w*_0_*-w*_5_ and two individual basic input data of *x*_*i*_ and *x*_*j*_, as the following function:


(2) $$\begin{array}{l} \phi_{n}=w_{0}+w_{1}x_{i}+w_{2}x_{j}+w_{3}x_{i}^{2}+w_{4}x_{j}^{2}+w_{5}x_{i}^{\;}x_{j}^{\;} \end{array}$$

The data set provided by non-linear map ϕ_*n*_ using the RSM provides the second-order non-linear relations with a linear correlation between input data as $x_{i}^{\;}x_{j}^{\;}$using Equation (2). ϕ_*n*_ *n* = 1,2,…, *m* has a similar dimension as well as PPV due to the use of RSM as the predictor of PPV. The last square estimator is applied to estimate the weights in Equation (2) as follows (66):


(3) $$\begin{array}{l} w={\left[P{\left(X\right)}^{T}P\left(X\right)\right]}^{-1}\left[P{\left(X\right)}^{T}\left\{\begin{array}{c} x_{i}\\ x_{j} \end{array}\right\}\right] \end{array}$$

where,


(4) $$\begin{array}{l} P\left(X\right)=\left[\begin{array}{cccc} 1\;x_{i,1}\;x_{j,1} & x_{i,1}^{2} & x_{j,1}^{2} & x_{i,1}^{\;}x_{j,1}^{\;}\\ 1\;x_{i,2}\;x_{j,2} & x_{i,2}^{2} & x_{j,2}^{2} & x_{i,2}^{\;}x_{j,2}^{\;}\\ \vdots \vdots \vdots & \vdots & \vdots & \vdots \\ 1\;x_{i,N}\;x_{j,N} & x_{i,N}^{2} & x_{j,N}^{2} & x_{i,N}^{\;}x_{j,N}^{\;} \end{array}\right] \end{array}$$

where i and j are the two input variables, and *N* represents the number of data points in the training phase. The data in the hidden nodes can be predicted using the weight. In the second process, using the *m*–input data provided by RSM, the SVR model is trained by the following relation (67):


(5) $$\begin{array}{l} y=b+\overset{N}{\underset{i=1}{\sum }}w_{i}K\left(\phi ,\phi_{i}\right) \end{array}$$

where *b* is bias and *K*(ϕ, ϕ_*i*_) represents the Kernel function. The Gaussian kernel function is commonly implemented as follows (68–71):


(6) $$\begin{array}{l} K\left(\phi ,\phi_{i}\right)=\exp\left(-0.5{\left(\phi ,\phi_{i}\right)}^{2}/\sigma^{2}\right) \end{array}$$

where σ denotes the parameter of kernel functions as σ > 0, which controls the smoothness of the kernel function. The weights of the *N*–feature data set in the SVR model are computed using the following model:


(7) $$\begin{array}{l} \begin{array}{l} Min\frac{{||w||}^{2}}{2}+C\overset{N}{\underset{i=1}{\sum }}\left(\xi_{i}+\xi_{i}^{*}\right)\\ S.t.\{\begin{array}{c} y_{i}-<w.K\left(x,x_{i}\right)>-b\leq \varepsilon +\xi_{i}\\ <w.K\left(x,x_{i}\right)>+b-y_{i}\leq \varepsilon +\xi_{i}^{*}\\ \xi_{i},\xi_{i}^{*}\geq 0 \end{array} \end{array} \end{array}$$

where $\xi_{i},\xi_{i}^{*}$ are the positive coefficients as errors, which are used to compute the errors of the predicted data using SVR (*w*.*K*(*x, x*_*i*_)+*b*) and observed data (*y*_*i*_);ε represents the ε-insensitive loss function to neglect the error less than ε. *C* is a positive factor. The SVR model is executed using parameters of ε, σ, and *C* which are given based on the trial-and-error method.

In addition, an RSM is a simple method to provide the non-linear polynomial relation (72). It is combined by SVR in the proposed learning approach. In an RSM, the data for input variables of SVR is predicted based on the least square method (73–75) in terms of the observed data, the two input data sets selected from *Mc, B/S, St, Di, E*, and *Vp*. The accuracy of predictions plays an essential role in designing and reliably evaluating the simulation of a complex engineering problem. The non-linear forms in the hidden nodes of the proposed method are considered by both Kernel relation and polynomial function. By applying two regression processes, the flexibility of the RSM is increased and then it can be applied to simulating and predicting non-linear problems such as the prediction of dissolved oxygen concentration (73), monthly pan evaporations (76), and the corroded burst pressure of pipelines (72). The *m*–data set provided by the RSM can be covered by the non-linear relations for complex problems, while the accuracy of prediction results using SVR in the next step is strongly improved by using the RSM to make non-linear input predictions. The proposed RSM-SVR machine learning model offers a high level of flexibility for predictions in non-linear problems. The regressed approach is presented by RSM-SVR based on the following steps:

1) Apply the RSM to the first calibrating stage as follows:

i) Give inputs (*x*_1_*, x*_2_*,…, x*_*n*_);
ii) Use two input variables from step (i) as input in the RSMs;
iii) Regress nodes of hidden layer 1 using the RSM by two input data sets provided by step (ii).

2) Apply the SVR to the second calibrating stage:

i) Give the input database to SVR from hidden layer 1;
ii) Select parameters for the SVR modeling procedure including ε, C, and σ;
iii) Train the SVR model using the input data set provided by RSM in the first calibrating stage.

## 3. Results

In this study, an MLR equation based on input parameters (*Mc, B/S, St, E, Vp*, and *Di*) and an output parameter (PPV) was constructed as follows:


(8) $$\begin{array}{r} PPV=30.489+0.019Mc-\frac{3.585B}{S}+0.0005St\\ -0.054E+0.0004Vp-0.074Di \end{array}$$

Four statistical factors, i.e., mean absolute error (MAE), root mean square error (RMSE), Nash and Sutcliffe efficiency (*NSE*), and agreement index (d), are used to compare the performance of the MLR, RSM, SVR, and RSM-SVR models. The accuracy and agreement of the predictions for all the models are evaluated using the statistical factors presented in Equations (9)–(12) for both the testing and training data sets. By computing RMSE and MAE, the lower values represent the more accurate models, while higher NSE and d values indicate higher agreement predictions for a model. Therefore, the lowest values for error comparative factors, i.e., RMSE and MAE, and the highest agreement factor, i.e., d and NSE coefficients, can be used to select a model with superior prediction from among the other modeling approaches (77–87).


(9) $$\begin{array}{l} RMSE=\frac{1}{N}\sqrt{\overset{N}{\underset{i=1}{\sum }}{\left[O_{i}-P_{i}\right]}^{2}} \end{array}$$

(10) $$\begin{array}{l} MAE=\frac{1}{N}\overset{N}{\underset{i=1}{\sum }}|O_{i}-P_{i}| \end{array}$$

(11) $$\begin{array}{l} NSE=1-\frac{\overset{N}{\underset{i=1}{\sum }}|O_{i}-P_{i}|}{\overset{N}{\underset{i=1}{\sum }}|O_{i}-\overset{\text{\_\_}}{O}|},-\infty <NSE\leq 1 \end{array}$$

(12) $$\begin{array}{l} d=1-\frac{\overset{N}{\underset{i=1}{\sum }}|O_{i}-P_{i}|}{\overset{N}{\underset{i=1}{\sum }}|O_{i}-\bar{O}|+|P_{i}-\bar{O}|},0<d\leq 1 \end{array}$$

where *N* is the number of data in training (65 points) and testing (25 points) phases, *O*_*i*_, *P*_*i*_, and $\bar{O}$ are observed data, predicted i-th data, and mean of the observed data, respectively, which are computed as follows:


(13) $$\begin{array}{l} \bar{O}=\frac{\overset{N}{\underset{i=1}{\sum }}O_{i}}{N} \end{array}$$

The experimental data sets used in this study are separated into two main categories: training and testing sets. The training points are used to build the relations using the MLR, RSM, and SVR models (with parameters as ε = 0.05, σ = 1.25, and *C* = 1,000), the RSM-SVR model (with SVR parameters: ε = 0.02, σ = 1.15, and *C* = 1,500), GA-SVR model (with GA parameters: number of generation = 500, number of population = 10, mutation = 0.025 and crossover percentage = 0.8) and PSO-SVR model (with PSO parameters: number of particles = 10, number of iterations = 500, inertia weight = 0.9, cognitive acceleration factor, i.e., C_1_ = 2; social acceleration factor, i.e., C_2_ = 2), while the testing points are used to validate the performance of the studied models in terms of accuracy. The errors of the predicted data points of *O*_*i*_−*P*_*i*_ (i.e., error = observed -predicted PPV) in the training and testing phases are compared to illustrate the robustness of the predictions of the studied models. Table 3 represents the comparative coefficients for both the training and testing data sets. Table 3 shows that the RSM-SVR model provided more accurate predictions than the other models for both the training and testing phases. In other words, this model obtained the lowest MAE and RMSE values and the highest d and NSE values. The proposed model was found to be as efficient as the SVR, and it strongly improved the computational *B* of the modeling process compared to the hybrid intelligent models of PSO-SVR and GA-SVR. The accuracy for decreasing the RMSE of the predicted PPV using the RSM-SVR model was enhanced by ~145%/650%, 90%/440%, 155%/670%, 74%/190%, and 75%/385% compared to the MLR, RSM, SVR, PSO-SVR, and GA-SVR models for training/testing, respectively. As can be seen, by applying a two-regression procedure provided by RSM in the first stage and SVR in the second one, the proposed method can significantly improve the accuracy of the perdition for the non-linear problem of predicting blast-induced ground vibrations. The hybrid optimization approaches integrating GA and PSO with SVR succeeded in further enhancing the capabilities of the predicted models compared to SVR and RSM.

**Table 3:** Comparative error and agreement factors.

**Methods**	**Train phase**				**Test phase**				**Time (s)**
	**MAE**	**RMSE**	**d**	**NSE**	**MAE**	**RMSE**	**d**	**NSE**	
MLR	1.707	2.225	0.859	0.719	3.429	3.977	0.616	0.220	2.38
RSM	1.218	1.605	0.900	0.800	2.361	3.073	0.715	0.463	3.62
SVR	1.688	2.300	0.863	0.722	3.503	4.114	0.623	0.203	6.21
PSO-SVR	0.661	0.868	0.946	0.891	1.992	2.227	0.787	0.524	484.83
GA-SVR	1.397	1.780	0.886	0.770	2.152	2.837	0.741	0.480	543.41
RSM-SVR	0.061	0.296	0.995	0.990	1.379	1.619	0.832	0.686	8.94

**Figure 3:**
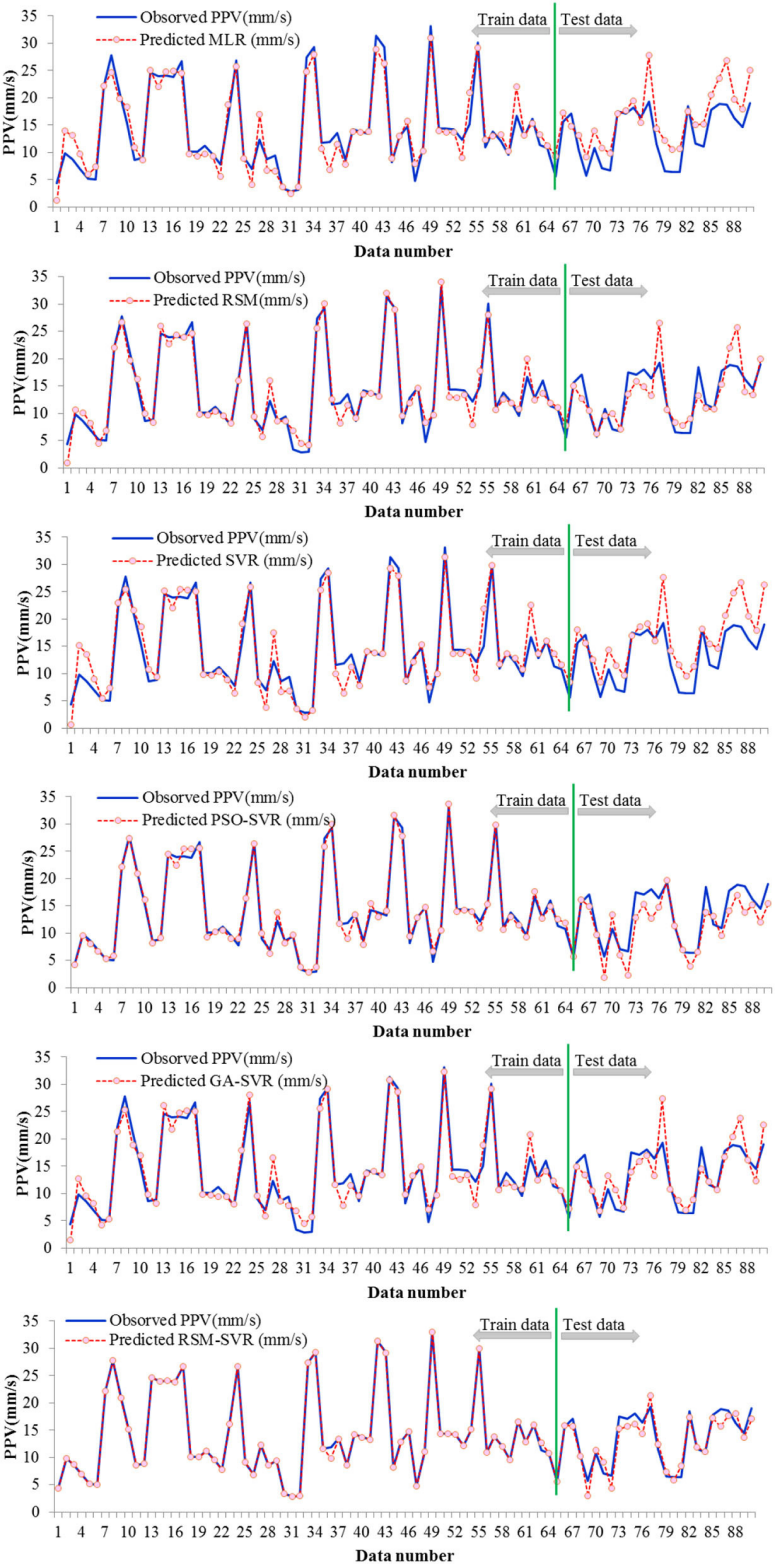
Comparison of predicted and observed data points of PPV for the developed models.

The values of PPV for both predicted and observed PPV in the training and testing phases are plotted in Figure 3 for all studied models. This figure clearly reveals that the RSM-SVR model showed a superior predictive ability compared to the PSO-SVR, GA-SVR, SVR, RSM, and MLR models. This was because a good agreement between the predicted and experimental PPV values was obtained, shown by the highest d and NSE values presented in Table 3.

**Figure 4:**
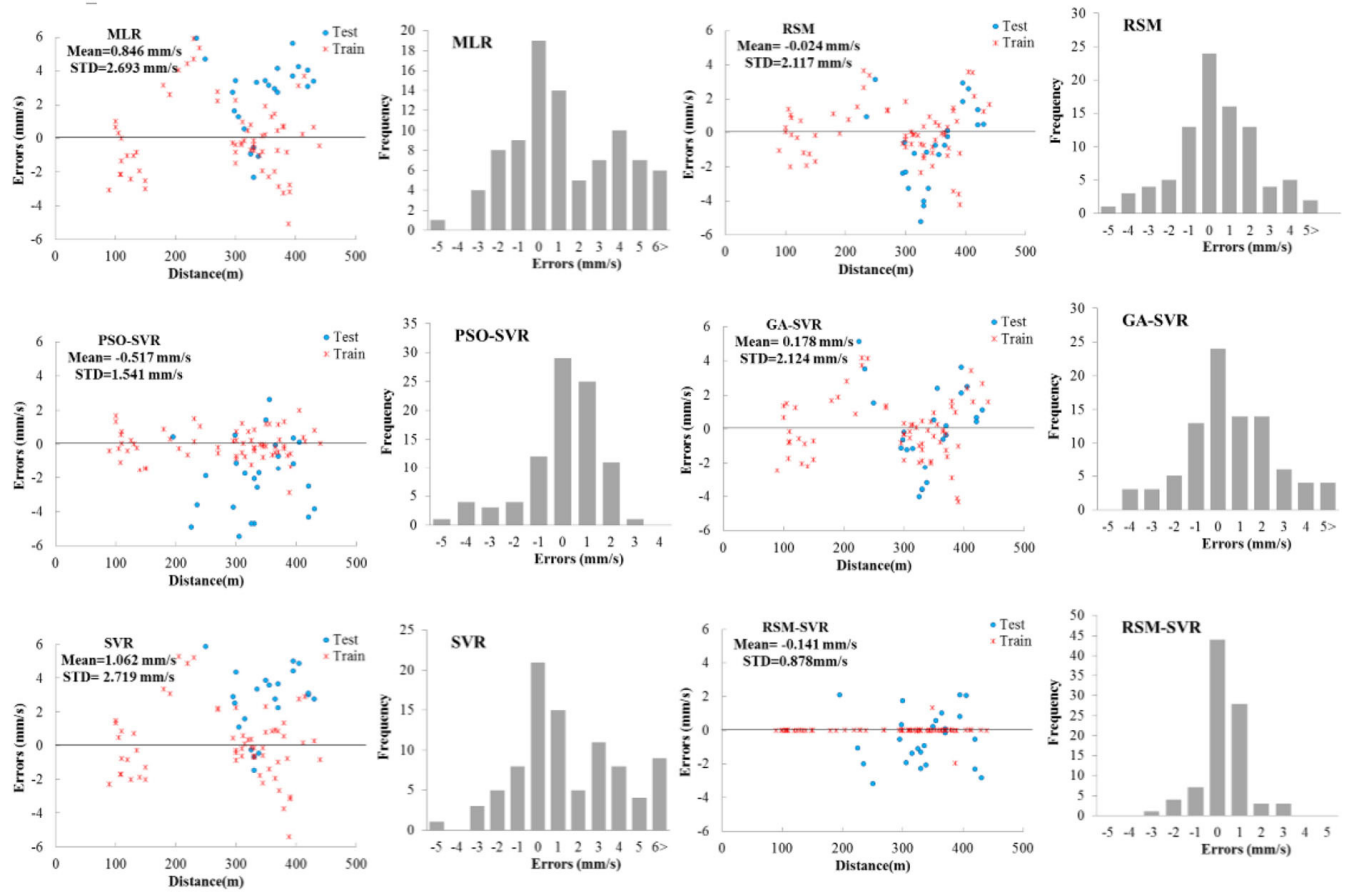
Errors (*PPV*_*exp*_-*PPV*_*Pre*_) of predictive models shown in bar diagrams, corresponding to distance.

The errors observed-predicted data points of PPV, i.e., *PPV*_*exp*_-*PPV*_*Pre*_, were evaluated for developed models. The bar diagrams of errors and the scatter points for both the training and testing data sets with respect to the input variables of distance are presented in Figure 4. As this figure shows, the standard divisions (STD) for errors were obtained as 2.693, 2.217, 1.541, 2.124, 2.719, and 0.878 mm/s for the MLR, RSM, PSO-SVR, GA-SVR, SVR, and proposed RSM-SVR models, respectively. The MLR, RSM, and SVR models presented the predicted data with the same scatter. The distributed formats of the errors for these models are presented in error bars; the PSO-SVR model is offered among the models studied. The proposed model shows the perfect predictions for PVV in the training phase for almost all data points and its error bar shows narrower distributed bars compared to the PSO-SVR. This means that the proposed model has high flexibility to provide the non-linear relations for complex engineering problems, while the existing modeling approach based on MLR, GA-SVR, and SVR provides an error bar scattered similar to the regression approaches RSM. The hybridization approach to regression using the proposed RSM-SVR and PSO-SVR models enhances the predictions of complex engineering problems such as the PPV estimation.

**Figure 5:**
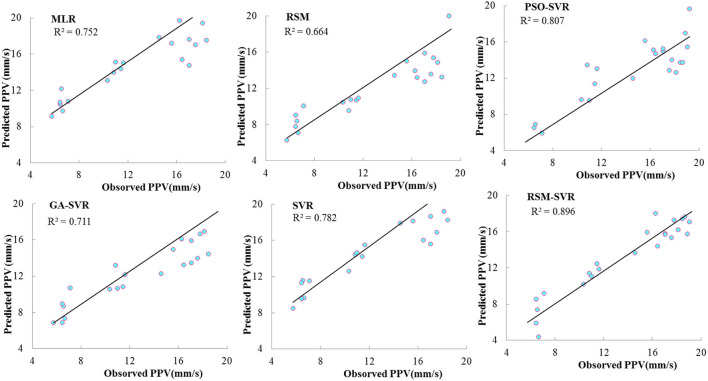
Scatterplot of predicted and observed PPV for test data set.

Figure 5 shows the scatterplots for the predicted data points in the testing set corresponding to the observed PPV. According to this figure, the PSO-SVR, SVR, and RSM-SVR models provided relations of higher non-linearity compared to the MLR models with higher correlation coefficients: *R*^2^ = 0.896 for RSM-SVR, *R*^2^ = 0.807 for PSO-SVR, *R*^2^ = 0.782 for SVR, the MLR with *R*^2^ = 0.752, and GA-SVR with *R*^2^ = 0.711. Consequently, it can be concluded that the flexible non-linear relations are provided based on two regression phases in the proposed models, while other comparative models are established based on one regression phase. The non-linear map using the RSM as input variables provides an acceptable input variable for the prediction of SVR in the proposed hybrid model for accurate predictions of PPV. The RSM shows the prediction results with the lowest accuracy, *R*^2^ = 0.664, while the non-linearity degree of the RSM is more than MLR but still does not show the acceptable flexibility for modeling this problem with less error than MLR. Due to the high flexibility in providing the non-linear relation, the accuracy of the proposed RSM-SVR model is strongly improved compared to the SVR, GA-SVR, and RSM models. The proposed model strongly improved the prediction performance of the RSM for this complex problem.

## 4. Discussion

**Figure 6:**
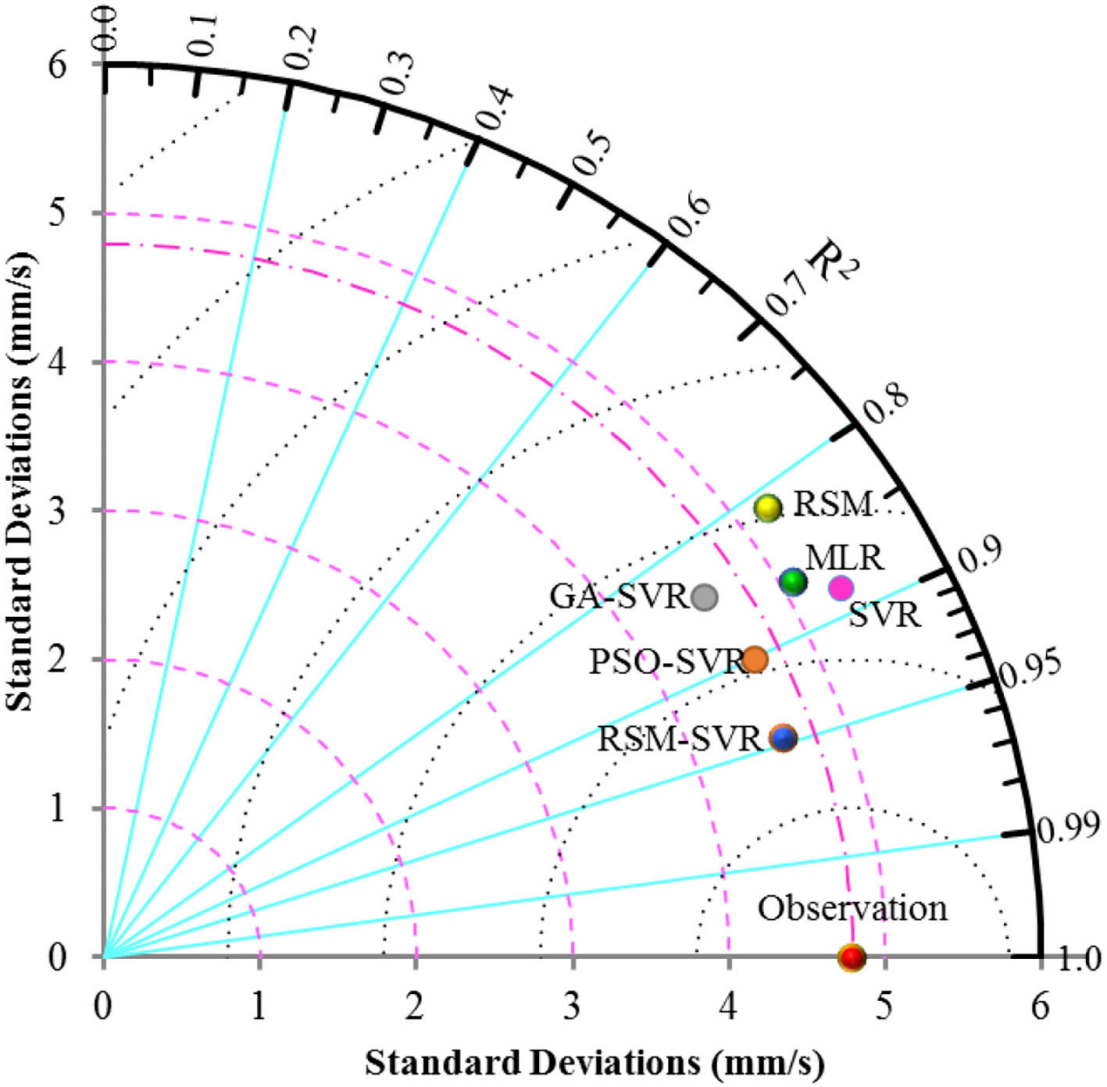
Taylor diagram for different models in the testing phase.

**Figure 7:**
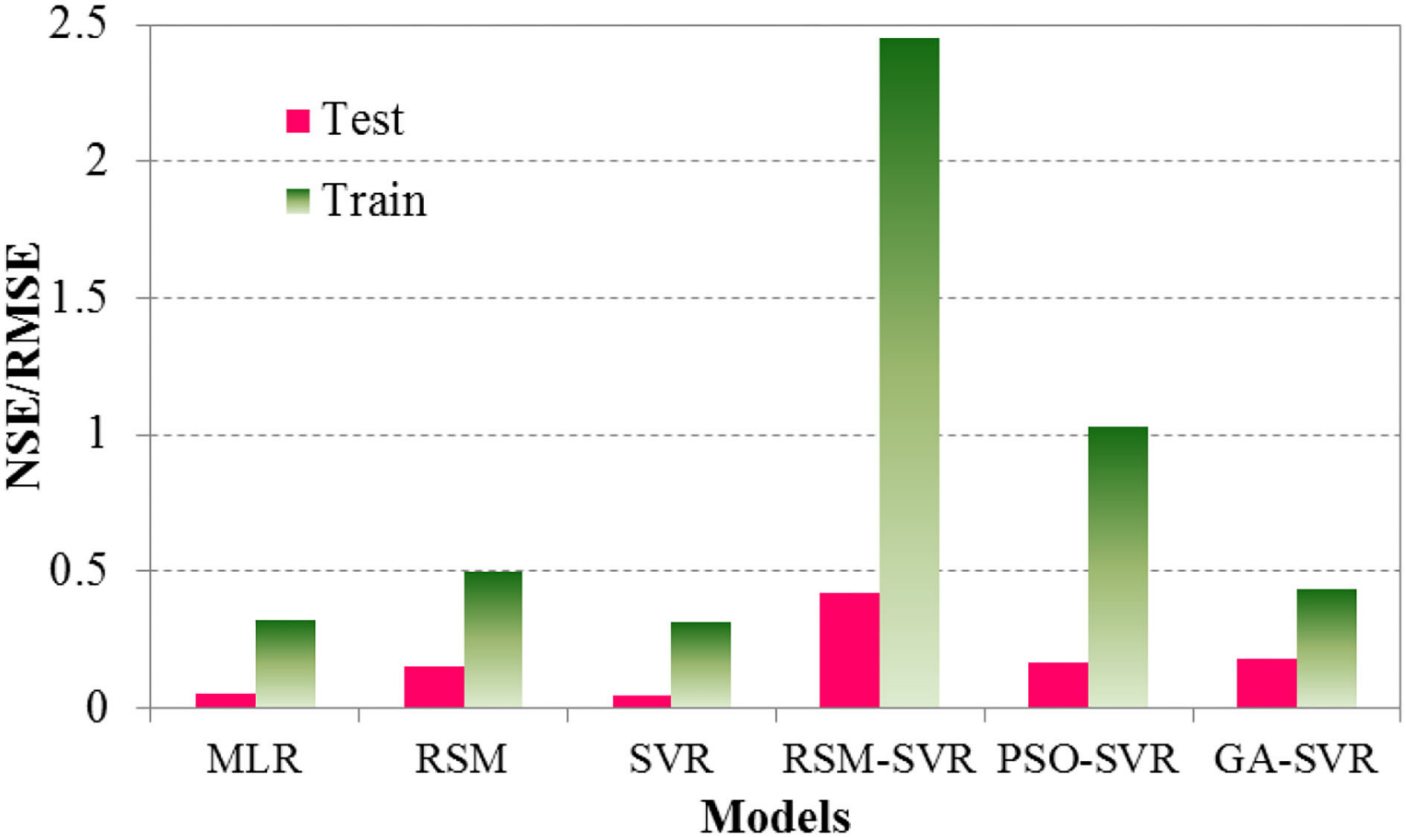
NSE/RMSE factors for different models in the training and testing phases.

To have a better discussion about the accuracy and agreement of the models, the Taylor diagram was used and the NSE/RMSE of the developed models were taken into consideration. The Taylor diagram is used by standard deviations and *R*^2^ of predicted results for different models can show the models performance. The NSE/RMSE is computed based on the comparative statistics, which can be used for measuring accuracy (i.e., RMSE) and agreement (i.e., NSE). The larger the NSE/RMSE value, the superior the performance of the model. The Taylor diagrams for the RSM, MLR, SVR, PSO-SVR, GA-SVR, and RSM-SVR models with observed data (represented by the red point on the horizontal line as observation) are presented in Figure 6, for the testing database. The NSE/RMSE results for different models in the testing and training data sets are shown in Figure 7.

Figure 6 and the NSE/RMSE ratios presented in Figure 7 indicate that the models have performed differently in accuracy and agreement. SVR offered higher agreement than MLR and the RSM, while it showed less agreement than the PSO-SVR, GA-SVR, and RSM-SVR models. MLR and the RSM-SVR showed close standard deviations compared to the others. The RSM-SVR offered more accurate predictions than the other models and it showed a higher agreement compared to the SVR, GA-SVR, RSM, and MLR models for the complex problem at hand. The optimization method for tuning the parameters of the hybrid SVR models (i.e., ε, *C*, and σ) of PSO-SVR can improve the capabilities of SVR models for this problem. By comparing the results represented by the Taylor diagram, the models were ranked from best to worst as follows: (1) RSM-SVR, (2) PSO-SVR, (3) GA-SVR, (4) SVR, (5) MLR, and (6) RSM.

The capabilities of the proposed hybrid SVR method in terms of both agreement and accuracy were enhanced using two regression procedures. The data handling set as input (which was provided by the RSM) was used for the training process of the SVR model, while the main objective for hybridizing the SVR with the optimization methods of PSO (PSO-SVR) and GA (GA-SVR) was to find the best parameters of the SVR model. The use of optimization algorithms in the PSO-SVR and GA-SVR models causes them to consume more computation time than the RSM-SVR model. By comparing the RSM-SVR model with hybrid intelligent models of PSO-SVR and GA-SVR, the main advantages of the RSM-SVR model is the use of only one process for the SVR model regression and also the use of the handling input data computed by RSM. In future research, the proposed model and the models hybridizing SVR with optimization methods can enhance the quality of prediction in complex engineering problems.

It is worth mentioning that the performance of a predictor is significantly related to the database used as well as the type of rock. The field investigated in this study is a granite quarry located in Malaysia. By reviewing the previous studies, it was found that Zhang et al. (47), Jahed Armaghani et al. (48), and Zhou et al. (88) have previously conducted studies on the prediction of PPV in granite quarries located in this country. Therefore, the performance of the proposed RSM-SVR presented in this study can be compared with that of the models developed in the aforementioned studies. According to Zhang et al. (47), the PPV was predicted by CHAID, RF, ANN, SVM, and CART models. Based on their results, the values of *R*^2^ obtained from CHAID, RF, ANN, SVM, and CART models were equal to 0.68, 0.83, 0.84, 0.85, and 0.56, respectively. In the study conducted by Jahed Armaghani et al. (48), the LS–SVM, GPR, MPMR, and PSO- PSO-ELM models were employed. They developed three autonomous groups of PSO (AGPSO) models in combination with ELM. They showed the values of *R*^2^ in the range of 0.8–0.89 for the MPMR, LS–SVM, GPR, PSO-ELM, AGPSO1–ELM, AGPSO2–ELM, and AGPSO3–ELM models, respectively. Furthermore, Zhou et al. (88) predicted PPV using five different gene expression programming (GEP) models, and in the best model, the value of *R*^2^ was equal to 0.88. The RSM-SVR model proposed in this study predicted PPV with an *R*^2^ of 0.896 (~ 0.9). A comparison with the results of the above-mentioned models indicates the effectiveness of the RSM-SVR model in predicting PPV.

In this study, a sensitivity analysis was also performed to check the level of input parameters' intensity on the output parameter (PPV) through the following equation from Yang and Zhang (89):


(14) $$\begin{array}{l} r_{ij}=\frac{\overset{n}{\underset{k=1}{\sum }}\left(y_{ik}\times y_{ok}\right)}{\sqrt{\overset{n}{\underset{k=1}{\sum }}{y_{ik}}^{2}\overset{n}{\underset{k=1}{\sum }}{y_{ok}}^{2}}} \end{array}$$

where *y*_*i*_ and *y*_*o*_ are the input and output parameters, respectively. The parameter with the highest *r*_*ij*_ has the highest effect on output. According to the results, the *r*_*ij*_ values for the *Di, Vp, E, St, B/S*, and *Mc* were 0.729, 0.891, 0.877, 0.838, 0.892, and 0.839, respectively. Therefore, the *Vp* and *B/S* were identified as the most effective parameters on PPV.

## 5. Conclusions

The accurate prediction of PPV is of high importance to safety issues in surface mines. This study aimed to propose a novel hybrid SC model, namely the RSM-SVR model. This model is structured using an RSM in the first calibrating process and an SVR in the second calibrating process to improve the accuracy of the PPV predictions. In addition, SVR, SVR-PSO, SVR-GA, RSM, and MLR models were also used in this study for comparison purposes. The above-mentioned models were developed to predict PPV in a granite quarry located in Johor, Malaysia. To this end, 90 blasting events were monitored and the values of *B, S, St, Mc, Di, E*, and *Vp* were precisely measured. In addition, the obtained PPV values for each blasting event were recorded. After constructing the models, four statistical factors, namely MAE, RMSE, NSE, and d were implemented to check the performance of the models. Finally, the following conclusions were drawn from this study:

- Experimental results demonstrated that the RSM-SVR model achieved the greatest evaluation criteria for *R*^2^ (0.896), MAE (1.379 mm/s), RMSE (1.619 mm/s), d (0.832), and NSE (0.686).
- Comparing the results, it was found that the RSM-SVR model provided a prediction of relatively higher accuracy than that of the PSO-SVR, GA-SVR, MLR, SVR, and RSM models.
- Hybridizing the SVR and an RSM is a powerful approach to solving prediction-based problems and has the capacity to be generalized to other fields.
- According to the sensitivity analysis, the *Vp* and *B/S*, were the most effective parameters on the PPV.

To end with, future research directions include, but are not limited to, the following: (1) applying the RSM-SVR model to other prediction problems in the fields of mining and geotechnical engineering; and (2) using other optimization algorithms such as the variable depth search algorithm, cultural algorithm, water flow-like algorithm, cyber swarm algorithm, water wave optimization, and jaguar algorithm to improve the SVR performance.

## Data availability statement

The original contributions presented in the study are included in the article/supplementary material, further inquiries can be directed to the corresponding author.

## Author contributions

Conceptualization and data curation: MH. Methodology and validation: BK and JP. Investigation: JP and BN. Writing—original draft preparation and writing—review and editing: BK, JP, RA, MH, MM, and BN. Supervision: MH and RA. Funding acquisition: MM. All authors have read and agreed to the published version of the manuscript.
